# Skin Color and Attractiveness Modulate Empathy for Pain: An Event-Related Potential Study

**DOI:** 10.3389/fpsyg.2021.780633

**Published:** 2022-01-04

**Authors:** Di Yang, Xiong Li, Yinya Zhang, Zuoshan Li, Jing Meng

**Affiliations:** ^1^Key Laboratory of Applied Psychology, Chongqing Normal University, Chongqing, China; ^2^School of Education, Chongqing Normal University, Chongqing, China; ^3^Faculty of Psychology, Southwest University, Chongqing, China; ^4^Key Laboratory of Basic Psychology, Southwest University, Chongqing, China

**Keywords:** empathy, pain, skin color, attractiveness, event-related potentials

## Abstract

Although racial in-group bias in empathy for pain has been reported, empathic responses to others’ pain may be influenced by other characteristics besides race. To explore whether skin color and attractiveness modulate empathy for pain, we recorded 24 participants’ reactions to painful faces from racial in-group members with different skin color (fair, wheatish, or dark) and attractiveness (more or less attractive) using event-related potentials (ERPs). Results showed that, for more attractive painful faces, dark skin faces were judged as less painful and elicited smaller N2 amplitudes than fair- and wheatish-skinned faces. However, for less attractive faces, there were no significant differences among the three skin colors. Our findings suggest that empathy for pain toward racial in-group members may be influenced by skin color and attractiveness.

## Introduction

Empathy refers to a complicated psychological construct that reflects the ability to understand and share others’ emotional states (Arditte Hall et al., 2018). When an individual observes pain or injury of others, they often perceive pain and negative experiences as their own (Levy et al., 2018). This ability is called empathy for pain (Han, 2018; Meng et al., 2019). Empathy helps individuals to avoid potential hazards and promotes empathic behavior in others (Shi et al., 2015; Webb et al., 2017).

The neurophysiological mechanisms underlying empathy for pain can be measured using event-related potentials (ERPs), which have high temporal resolution and can be used in experimental paradigms to record some subjective responses (Cascio et al., 2015; Hu and Iannetti, 2019). Empathic neural responses to others’ pain have been observed in several ERP components following the onset of painful pictures (Jing et al., 2017; Han, 2018; Luo et al., 2018; Meng et al., 2019). For example, an early ERP component (N2) over the frontal-central area of the cortex is related to affective empathy to others’ pain and positively correlates with participants’ personal negative emotional reactions (Mokhtari et al., 2020). What’s more, prior studies found that N2 amplitudes might be the key component in empathy and could predict adults’ affective empathy, with greater N2 amplitudes correlated with more immediate affective empathy (Kerr-Gaffney et al., 2019). Later ERP components [e.g., P3 and late positive complex (LPC), over the central-parietal cortex] represents cognitive empathy and has been shown to correlate with pain intensity judgment of others’ pain (Chen and Liu, 2016; Xia et al., 2016; Meng et al., 2019).

In addition, as principal ERP components elicited after task-relevant visual stimuli, Hong et al. (2016) similarly found the two important time temporal stages of processing painful attractive and unattractive faces pictures (Hong et al., 2016), including an early negativity (N2) around 200–300 ms after the onset of a stimulus and late LPC which peak at the parietal electrodes later than 300 ms post-stimulus. Additionally, researchers have conducted numerous ERP studies on faces with race characteristics. One representative ERP components have been investigated in these studies, namely N170. The N170 is an early negative-going potential over occipito-temporal regions usually peaking at around 170 ms post-stimulus (Beckes et al., 2013; Recio et al., 2014), and is considered to be an indicator of automatic or unconscious processing of facial features (Hajcak et al., 2010). Some researchers found the N170 amplitudes elicited by viewing faces of other races (vs. own-race) were larger (Ito and Urland, 2005; Stahl et al., 2008) which suggests that the N170 may represent social categorization processes.

Although people use various social cues for racial categorization, skin color is one of the most salient race-related phenotypic features (Ebner et al., 2011). Skin color is considered a racial feature that helps to rapidly identify whether an individual belongs to a certain race (Pereira et al., 2019). Indeed, previous studies have shown that distinguishing racial identity according to others’ skin color occurs spontaneously and automatically affects subsequent interpersonal thoughts, feelings, and behavior in observers (Nguyen et al., 2018; Deska et al., 2020).

Racial in-group bias in empathy has been widely reported in previous studies, shown by greater empathy for pain toward racial in-group members compared with that of racial out-group members (e.g., Han, 2018; Luo et al., 2018). One study showed that when Chinese participants were presented with painful facial pictures of Chinese (wheatish skin) and Caucasian (fair skin) models, their empathic neural responses to painful facial pictures of Caucasian models were lower than those of Chinese models (Feng et al., 2015). A similar pattern was found by Fabi and Leuthold (2018), where they compared electroencephalogram (EEG) responses of Caucasian participants (fair skin) to painful pictures of fair- and dark-skinned hands and found that Caucasian participants showed decreased empathic responses toward dark-skinned hands than fair-skinned hands (Fabi and Leuthold, 2018). These studies suggest that individuals exhibit greater empathic responses to racial in-group members’ pain than to racial out-group members’ pain. This effect is explained by racial in-group bias in empathy (Avenanti et al., 2010; Magariño et al., 2020). However, racial identity used in these studies was mainly represented by the skin color of parts of the body (e.g., faces and hands), and models with a similar skin color as participants were perceived as racial in-group members, whereas those with dissimilar skin color were perceived as racial out-group members. Furthermore, it is possible that skin color also represents physical fitness and attractiveness of an individual (Stepanova and Strube, 2012; Niesta Kayser et al., 2016; Visconti et al., 2018; Freitas et al., 2020). Thus, others’ skin color may play a crucial role that may currently be underestimated. It remains unclear whether empathic responses to others’ pain could be influenced by the skin color of racial in-group members.

Skin color, especially facial skin color, plays an important role in judgments of physiological health, which include fitness, immunity, and fertility (Bixley et al., 2018). There have been numerous studies that have suggested that individuals’ perceptions of physical fitness are influenced by skin color (Carrito et al., 2016; Dias, 2020), and physical fitness has important implications for resisting potential threat and harm (Ogunjimi et al., 2020). One study showed that when participants were asked to select the healthiest person from photographs of individuals with different skin color, they consistently chose dark skin over fair or wheatish skin (Cairns et al., 2020). Furthermore, individuals with slightly dark skin (which may indicate more efficient blood circulation) were considered more attractive and healthy (Jones, 2018). This may be because individuals with dark skin are considered to have low risk of sunburn and skin diseases (Stepanova and Strube, 2012; Desai et al., 2020; Freitas et al., 2020).

The “beauty-is-good” stereotype (Little et al., 2006) supposes that facial attractiveness is a marker of biological quality that signals fertility and health and that it plays a significant role in interpersonal interactions in daily life (Nakamura and Watanabe, 2020). To date, there have been no consistent conclusions regarding the influence of attractiveness on empathy for pain. One study showed that attractiveness facilitates empathy for pain, whereby greater empathic responses were elicited for more attractive than less attractive faces (Meng et al., 2020). However, another study revealed that physical attractiveness inhibits children’s empathy for pain (Fisher and Ma, 2014). Therefore, to investigate the effect of others’ skin color on empathic responses to others’ pain and the interaction between skin color and attractiveness, we considered the modulation effect of both skin color and attractiveness in the present study.

Based on previous findings showing that individuals with dark skin are perceived as healthy and having better physical fitness (Ogunjimi et al., 2020) and the “beauty-is-good” stereotype (Little et al., 2006), we hypothesized that empathy for pain would be influenced by others’ attractiveness and skin color and that both behavioral and neural responses to more attractive and dark-skinned individuals in pain would be inhibited.

## Materials and Methods

Twenty-four adults (13 women) from the Chongqing Normal University participated in this study as paid volunteers. None of the participants had been previously diagnosed with a psychiatric, medical, or neurological disorder. All participants were right-handed Chinese adults between the ages of 18 and 24 years [mean = 21.8 years, standard deviation (*SD*) = 2.4 years]. Written informed consent was provided by all participants prior to participation in the experiment in accordance with the Declaration of Helsinki, and all procedures were approved by Chongqing Normal University research ethics committee. The procedures were performed in accordance with ethical guidelines and regulations.

After conducting the experiment, a *post hoc* power analysis using Gpower 3^1^ (Faul et al., 2007) was conducted using a conservative average of the moderate effect sizes from previous sharing empathy for pain studies (within factors, *F*-test, Cohen’s *d* = 0.79–0.44; Rütgen et al., 2015a,b). We used sample size of 24 participants to calculate a power of 1−ß = 0.99 at a standard error probability of α = 0.05 with moderate effect size of *d* = 0.4.

### Stimuli

**FIGURE 1:**
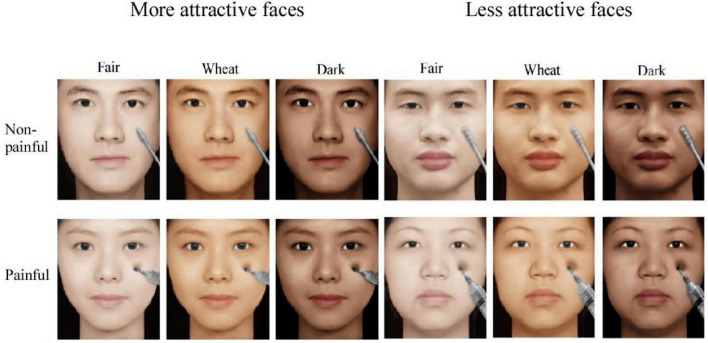
Examples of pictures used in the study. Examples of more (left panel) and less (right panel) attractive faces and painful (top panel) and non-painful (bottom panel) faces with fair, wheatish, and dark skin. Pictures were revised from a picture database that had been validated and used in previous studies Li et al. (2020) and Meng et al. (2020).

The stimuli (see Figure 1 for examples) were 480 digital pictures of Chinese faces, which were revised from a picture database that had been validated and used in previous studies (Li et al., 2020; Meng et al., 2020). The database comprised pictures of 40 more attractive faces (20 female faces and 20 male faces) and 40 less attractive faces (20 female faces and 20 male faces). Painful pictures depicted the model having a syringe needle penetrating their cheek, and non-painful pictures depicted a soft object (Q-tip) gently touching the model’s cheek. The skin color of each face was transformed into three different skin colors (fair, wheatish, and dark) using the Adobe Photoshop CS2 (Adobe Systems Incorporated, CA, United States) software. Luminance, contrast, and color were matched across painful and non-painful pictures. Moreover, to reduce interference caused by repetitive stimuli, all pictures were mirror flipped once.

Before the experiment, skin color (1 = fair, 5 = wheatish, and 9 = dark), attractiveness (1 = not at all attractive, 9 = most attractive), and emotional valence (1 = very happy, 5 = neutral, and 9 = very unhappy) of the pictures were assessed using a 9-point Likert scale by 51 undergraduate students (25 women, aged 18–26 years, mean = 24.22 years, *SD* = 3.4 years) who did not participate in the experiment. Detailed descriptive statistics of this assessment are summarized in Supplementary Table 1.

### Experimental Procedure

**FIGURE 2:**
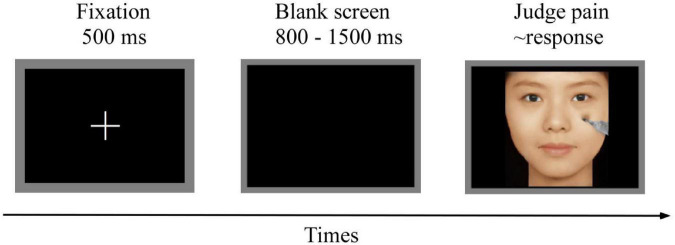
Flowchart describing the procedure of the experiment.

Participants were seated in a quiet and comfortable room with an ambient temperature of ∼23°C. Participants were instructed to determine whether the model in picture was experiencing pain. As shown in Figure 2, at the start trial, a 500 ms white fixation cross was presented on a black screen, followed by a blank black screen that was presented for 800–1,500 ms. A picture was then presented, and participants were instructed to respond as accurately and as quickly as possible by pressing a key (either “1” or “2”) to judge whether the presented face in the picture was experiencing pain. The keys pressed were counterbalanced across participants to control for order effects. The picture disappeared from the screen as soon as the participant responded. If the participant did not respond, the next trial was automatically carried out after 3,000 ms. The order of picture presentation was randomized. Presentation of pictures was controlled using the E-Prime 3.0 software (Psychology Software Tools, PA, United States). The entire experimental procedure comprised four blocks, with 240 trials per block and an inter-trial interval of 500 ms. Each picture was presented once during the experiment. A training session was conducted before the formal experiment to allow participants to familiarize themselves with the experimental procedure. EEG data were recorded throughout the experimental procedure.

Following the EEG recording session, participants were asked to rate each picture based on four attributes on a 9-point Likert scale, which included pain intensity (1 = no sensation, 4 = pain threshold, and 9 = unbearable pain), skin color (1 = fair, 5 = wheatish, and 9 = dark), and attractiveness (1 = not at all attractive, 9 = more attractive) of the model in the pictures, and their subjective emotional reaction (1 = very unhappy, 5 = neutral, and 9 = very happy).

### Electroencephalogram Recording

Electroencephalogram data were recorded from 64 scalp sites using tin electrodes mounted on an actiCHamp system (Brain Vision LLC, Morrisville, NC, United States; bandpass: 0.01–100 Hz; sampling rate: 1,000 Hz). The electrode on the medial frontal aspect was used as the ground electrode. All electrode impedances remained below 5 kΩ.

### Electroencephalogram Data Analysis

Electroencephalogram data were pre-processed and analyzed using MATLAB R2014a (MathWorks, United States) and the EEGLAB v13.6.5b toolbox (Delorme and Makeig, 2004). We used left-right mastoids as analyzing reference. Continuous EEG signals were bandpass filtered (0.01–30 Hz). Time windows of 200 ms before and 800 ms after the onset of stimuli were extracted from continuous EEG data, and the extracted window was baseline-corrected by a 200 ms time interval prior to stimulus onset. EEG epochs were visually inspected and trials containing significant noise from gross movements were removed. Electro-oculogram artifacts were corrected using an independent component analysis (ICA) algorithm (Jung et al., 2001). Epochs with amplitude values exceeding 80 μV at any electrode were excluded from the presented average. In our experimental tasks, EEG signals were impaired by participants’ movement, violent blinking, electromyography, or other interference during the experiment. We excluded some of bad trials. These excluded trials only constituted 6 ± 4.1% of the total number of trials.

We confirmed scalp topographies in both single-participant and group-level ERP waveforms. The ERP components selected in this study included early components (N1, P2, and N2) and late components (P3 and LPC) based on previously studies of empathy for pain (Sessa and Meconi, 2015; Hu and Iannetti, 2016; Meng et al., 2020). We also included N170, which is induced by face stimuli (Itier and Taylor, 2004). ERP components were extracted from following electrode sites: N1 (FCz, FC1, FC2, Cz, C1, and C2) within N1 latency intervals of 100–120 ms; P2 and N2 (AFz, AF3, AF4, Fz, F1, F2, FCz, FC1, and FC2) within P2 latency intervals of 180–200 ms and N2 latency intervals of 200–220 ms; P3 and LPC (CPz, CP1, CP2, Pz, P1, P2, POz, PO3, and PO4) within P3 latency intervals of 290–310 ms and the LPC was extracted within a time window of 400–600 ms; N170 (P7, P8, PO7, and PO8) within latency intervals of 160–180 ms.

To obtain genuine neural responses of empathy for pain, differential ERP waves were also used in the present study, which were obtained by subtracting the ERP waves of non-painful pictures from those of painful pictures (Ibáñez et al., 2011; Meng et al., 2012; Cui et al., 2016). Amplitudes of differential ERP waveforms were calculated at the same electrode sites and time windows as the original ERP components (i.e., N1, N2, P2, P3, LPC, and N170). Amplitudes of differential ERP waveforms were described as D_(ERP component); for example, D_N2 = N2 amplitude of painful pictures minus N2 amplitude of non-painful pictures.

### Statistical Analysis

#### Behavioral Data

Behavioral data, which included accuracies (ACCs), reaction times (RTs), and subjective ratings of pictures (i.e., pain intensity, attractiveness, skin color, and subjective emotional reaction ratings), were compared using a three-way repeated-measures analysis of variance (ANOVA), with within-participant factors of “pain” (painful, non-painful), “attractiveness” (more attractive, less attractive), and “skin color” (fair, wheatish, and dark). For significant interaction effects (*p* < 0.05), we performed simple effect analyses. The *p*-values of the main and interaction effects were corrected using the Greenhouse-Geisser method (Jessen and Kotz, 2011).

#### Event-Related Potential Data

Amplitudes of differential ERP waveforms between painful and non-painful pictures were compared using two-way repeated-measures ANOVA, with within-participant factors of “attractiveness” (more attractive, less attractive) and “skin color” (fair, wheatish, and dark). For significant interaction effects (*p* < 0.05), we performed simple effect analyses. The *p*-values of the main and interaction effects were corrected using the Greenhouse-Geisser method (Jessen and Kotz, 2011).

## Results

### Behavioral Data

**FIGURE 3:**
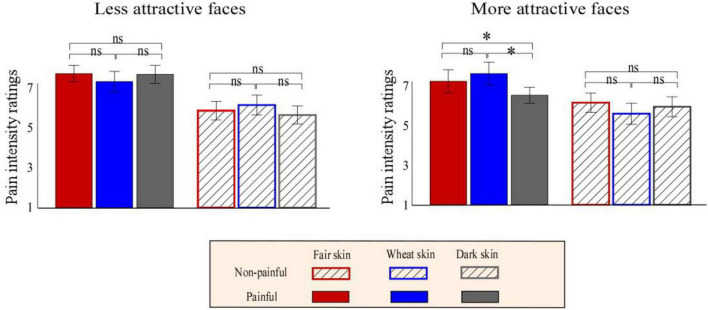
Bar charts of pain intensity ratings. Bar charts of the more attractive (right panel) or less attractive (left panel) faces with fair (red), wheat (blue), and dark (gray) skin with non-painful (linear) or painful (solid) cues. Data are expressed using means ± standard error of the mean. ns: *p* > 0.05, *p < 0.05, ^**^p < 0.01, ^***^p < 0.001.

The descriptive and statistical analysis results of the behavioral data are shown in Table 1 and Supplementary Table 2, respectively. Pain intensity ratings were modulated by the main effect of “pain” (*F*_1, 23_ = 153.66, *p* < 0.001, η_*p*_^2^ = 0.87), which showed that participants judged painful pictures as more painful than non-painful pictures (painful: 6.07 ± 0.46, non-painful: 4.33 ± 0.24). Pain intensity ratings were significantly modulated by the interaction of “pain,” “attractiveness,” and “skin color” (*F*_2, 22_ = 6.39, *p* = 0.005, η_*p*_^2^ = 0.22). Simple effects analyses indicated that for more attractive painful faces, dark-skinned faces were judged as less painful than fair-skinned (dark: 6.13 ± 0.22, fair: 6.92 ± 0.28; *F*_2, 22_ = 5.79, *p* = 0.024, η_*p*_^2^ = 0.20) and wheatish-skinned (wheatish: 7.35 ± 0.32; *F*_2, 22_ = 8.34, *p* = 0.008, η_*p*_^2^ = 0.27) faces. Pain intensity ratings did not differ between the three kinds of skin faces in the other conditions (*p* > 0.05 for all comparisons; Figure 3).

**TABLE 1:** Summary of repeated-measure analysis of variance (ANOVA) results of behavioral data.

	RT			ACC			Pain intensity rating			Attractive rating			Skin color rating			Emotional reaction		
	*F*	*P*	η_*p*_^2^	*F*	*P*	η_*p*_^2^	*F*	*P*	η_*p*_^2^	*F*	*P*	η_*p*_^2^	*F*	*P*	η_*p*_^2^	*F*	*P*	η_*p*_^2^
Pain	2.22	0.150	0.09	**8.92**	**0.007**	**0.28**	**153.66**	<**0.001**	**0.87**	1.18	0.290	0.05	3.37	0.079	0.13	**20.07**	<**0.001**	**0.54**
Attractiveness	0.11	0.918	<0.01	**9.18**	**0.006**	**0.29**	2.39	0.136	0.09	**9.48**	**0.005**	**0.29**	3.24	0.085	0.12	**40.09**	<**0.001**	**0.70**
Skin color	0.82	0.447	0.03	1.07	0.350	0.05	1.31	0.279	0.05	0.04	0.958	<0.01	**205.32**	<**0.001**	**0.89**	0.23	0.787	0.01
Pain × Attractiveness	0.25	0.622	0.01	2.26	0.146	0.09	0.36	0.080	0.13	0.10	0.753	<0.01	0.04	0.846	<0.01	1.46	0.244	0.08
Pain × Skin color	**5.79**	**0.006**	**0.20**	0.14	0.862	0.01	0.34	0.709	0.02	0.25	0.762	0.01	1.18	0.31	0.05	0.26	0.771	0.02
Attractiveness × Skin color	1.61	0.210	0.07	0.36	0.680	0.02	0.44	0.645	0.02	0.72	0.494	0.03	2.52	0.102	0.10	1.08	0.349	0.06
Pain × Attractiveness × Skin color	1.76	0.183	0.07	0.87	0.410	0.04	**6.39**	**0.005**	**0.22**	1.37	0.264	0.06	2.03	0.153	0.08	1.05	0.353	0.06

*Statistic results were obtained using three-way repeated measures ANOVA of “pain,” “attractiveness,” and “skin color.”*

*Significant comparisons (p < 0.05) were shown in boldface.*

Attractive ratings were significantly modulated by the main effect of “attractiveness” (*F*_1, 23_ = 9.48, *p* = 0.005, η_*p*_^2^ = 0.29), which indicated that participants judged more attractive faces as having higher attractiveness than less attractive faces (more attractive: 5.62 ± 0.13, less attractive: 5.13 ± 0.08). Skin color ratings were modulated by “skin color” (*F*_2, 22_ = 205.32, *p* < 0.001, η_*p*_^2^ = 0.89), which suggested that participants were able to accurately judge the three skin colors (fair: 3.51 ± 0.11, wheatish: 4.82 ± 0.13, and dark: 6.67 ± 0.09). Subjective emotional reactions were modulated by the main effects of “pain” (*F*_1, 23_ = 20.07, *p* < 0.001, η_*p*_^2^ = 0.54) and “attractiveness” (*F*_1, 23_ = 40.09, *p* < 0.001, η_*p*_^2^ = 0.70), which indicated that participants expressed more negative emotions to painful pictures than to non-painful pictures (painful: 4.71 ± 0.08, non-painful: 5.15 ± 0.06) and more positive emotions to the more attractive faces relative to the less attractive faces (less attractive: 4.58 ± 0.08, more attractive: 5.29 ± 0.06). No other main effects or interactions were significant (*p* > 0.05 for all comparisons).

Reaction times were significantly modulated by the interaction of “pain” and “skin color” (*F*_2, 22_ = 5.79, *p* = 0.006, η_*p*_^2^ = 0.20). For painful pictures, participants judged dark-skinned faces slower than they judged wheatish-skinned (dark: 674.75 ± 24.32 ms, wheatish: 692.01 ± 28.09 ms; *F*_2, 22_ = 5.55, *p* = 0.027, η_*p*_^2^ = 0.19) and fair-skinned (fair: 694.74 ± 27.86 ms; *F*_2, 22_ = 5.72, *p* = 0.025, η_*p*_^2^ = 0.20) faces. No differences were found in any of the other conditions (*p* > 0.05 for all comparisons).

Accuracies were modulated by the main effects of “pain” (*F*_1, 23_ = 8.92, *p* = 0.007, η_*p*_^2^ = 0.28) and “attractiveness” (*F*_1, 23_ = 9.18, *p* = 0.006, η_*p*_^2^ = 0.29). Participants judged painful pictures less accurately than they judged non-painful pictures (painful: 97.7 ± 6.3%, non-painful: 98.5± 5.4%) and judged more attractive faces less accurately than they judged less attractive faces (more attractive: 97.9 ± 5.7%, less attractive: 98.35 ± 4.6%). No other significant main effects or interactions were found (*p* > 0.05 for all comparisons).

### Event-Related Potential Data

**FIGURE 4:**
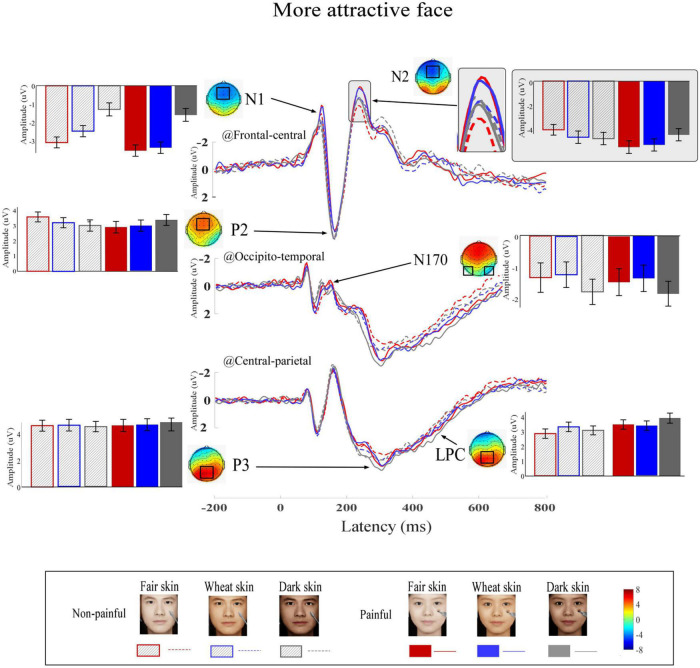
Event-related potential (ERP) waveforms and scalp topography distributions to more attractive faces. ERP waveforms, bar charts, and scalp topographies elicited by the more attractive faces with fair (red), wheat (blue), and dark (gray) skin. These pictures had either non-painful (linear) or painful (solid) cues. Electrodes used to estimate ERP amplitudes are marked by black squares on their respective topographic distributions. Data in the bar charts are ERP amplitudes expressed as means ± standard error of the mean.

**FIGURE 5:**
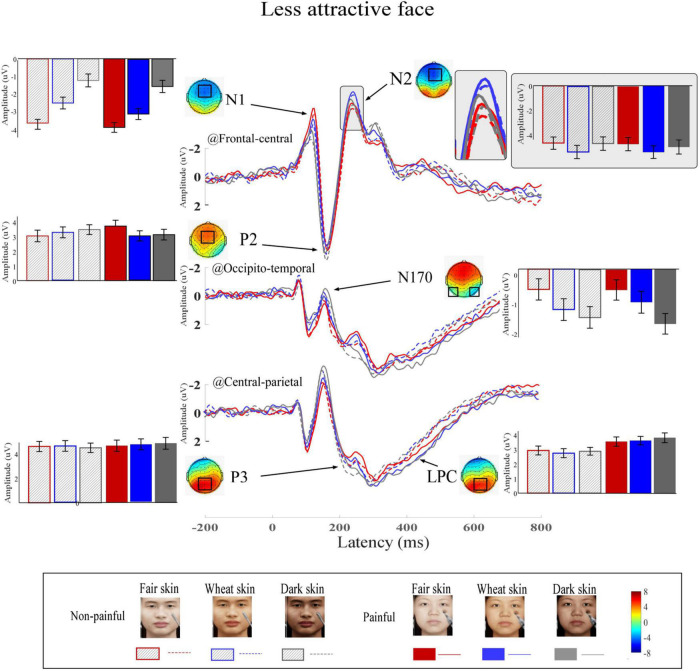
Event-related potential (ERP) waveforms and scalp topography distributions to less attractive faces. ERP waveforms, bar charts, and scalp topographies elicited by the less attractive faces with fair (red), wheat (blue), and dark (gray) skin. These pictures had either non-painful (linear) or painful (solid) cues. Electrodes used to estimate ERP amplitudes are marked by black squares on their respective topographic distributions. Data in the bar charts are ERP amplitudes expressed as means ± standard error of the mean.

Grand average ERP waveforms and scalp topographies of painful and non-painful pictures with different skin colors are shown in Figure 4 (high attractiveness faces) and Figure 5 (low attractiveness faces). These pictures elicited N1, N2, and P2 over frontal-central electrodes, N170 over occipito-temporal electrodes, and P3 and LPC at central-parietal electrodes.

**FIGURE 6:**
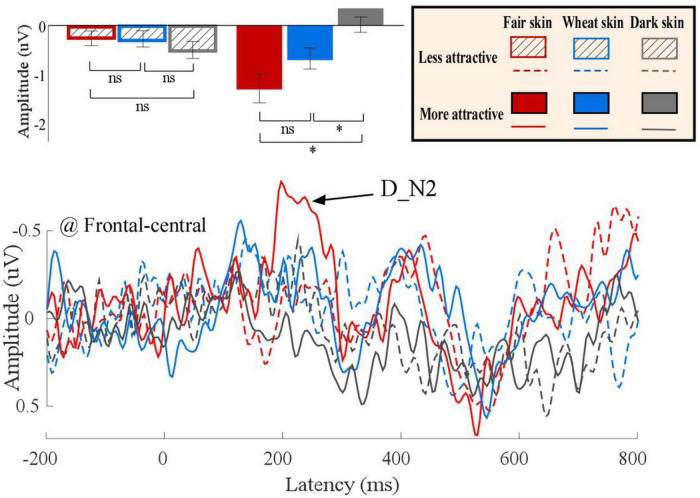
Differential event-related potential (ERP) waveforms between painful and non-painful pictures. Differential ERP waveforms (bottom panel) of more (solid) and less (dotted) attractive faces with fair (red), wheat (blue), and dark (gray) skin. The D_N2 amplitudes are shown in bar charts and are expressed as means ± standard error of the mean (top panel). ns: *p* > 0.05, *p < 0.05, ^**^p < 0.01, ^***^p < 0.001.

Differential ERP waveforms between painful and non-painful pictures are shown in Figure 6. Amplitudes of D_N2 were significantly modulated by the interaction of “skin color” and “attractiveness” (*F*_2_,_22_ = 3.69, *p* = 0.036, η_*p*_^2^ = 0.14). Simple effects analyses indicated that for more attractive faces, D_N2 amplitudes to dark-skinned faces (0.32 ± 0.33 μV) were significantly less negative (smaller amplitudes) than to fair- (−1.37 ± 0.49 μV, *F*_1, 23_ = 9.83, *p* < 0.005, η_*p*_^2^ = 0.29) and wheat-skinned (−0.64 ± 0.37 μV, *F*_1, 23_ = 5.22, *p* < 0.032, η_*p*_^2^ = 0.19) faces. There were no differences between the three skin colors for the less attractive faces (*F*_2, 22_ = 0.82, *p* = 0.092, η_*p*_^2^ = 0.01). No other main effects or interactions were found (*p* > 0.05 for all comparisons). Statistical analysis results are summarized in Table 2 and detail statistic results of amplitudes of the dominant ERP components were summarized in Supplementary Table 3.

**TABLE 2:** Summary of statistical analyses of amplitudes of differential event-related potential (ERP) waveforms.

	Attractiveness			Skin color			Attractiveness × Skin color		
	*F*	*P*	η_*p*_^2^	*F*	*P*	η_*p*_^2^	*F*	*P*	η_*p*_^2^
D_N1	0.72	0.405	0.30	1.85	0.171	0.07	0.15	0.841	0.01
D_N170	0.60	0.445	0.03	0.70	0.498	0.03	2.04	0.151	0.08
D_N2	0.50	0.486	0.02	1.95	0.154	0.08	**3.69**	**0.036**	**0.14**
D_P2	0.36	0.557	0.02	0.17	0.831	0.01	0.62	0.533	0.03
D_P3	3.48	0.074	0.13	0.86	0.428	0.04	0.39	0.649	0.02
D_LPC	2.01	0.170	0.08	1.03	0.360	0.04	0.93	0.400	0.04

*Summary of statistical analyses results of amplitudes of differential ERP waveforms between painful and non-painful pictures. Amplitudes of differential ERP waveforms were described as D_(ERP component); for example, D_N2 = N2 amplitude of painful pictures minus N2 amplitude of non-painful pictures. Results were obtained using repeated measures analysis of variance (ANOVA) with the within-participant of “attractiveness” and “skin color.”*

*Significant (p < 0.05) comparisons are indicated in boldface.*

## Discussion

The present study explored whether empathic responses to others’ pain are affected by others’ skin color and attractiveness. Our results showed that dark-skinned faces were judged as less painful and elicited smaller N2 amplitudes than fair- and wheatish-skinned faces. However, this effect was specific to more attractive painful faces, and not to less attractive faces. These results suggested that empathy for pain to more attractive people may be modulated by skin color and that empathic responses to more attractive dark-skinned painful faces may be inhibited.

The behavioral data analysis showed that participants expressed higher pain intensity ratings and more negative emotional reactions toward painful faces than toward non-painful faces. These results are in line with previous studies using painful pictures that exhibited injuries of the hands and feet (Chen et al., 2012; Fabi and Leuthold, 2017), faces (Meng et al., 2020) as well as painful expressions (Jie et al., 2017). Thus, our findings suggest that in the present study, participants’ affective and cognitive empathy was successfully elicited by the stimuli. In addition, consistent with previous findings that more positive emotional reactions are evoked by more attractive faces than they are by less attractive faces (Shang et al., 2018; Wang et al., 2018), our findings confirmed that emotional reactions were significantly modulated by the main effect of “attractiveness.”

Consistent with a previous ERP study of empathy for others’ facial pain (Meng et al., 2020), in our study, others’ painful faces elicited larger ERP amplitudes than did non-painful faces, which included the frontal-central N1 and the central-parietal P3 and LPC. Given that the N1 is thought to reflect early bottom-up processes, and the P3 and LPC are thought to be linked to top-down cognitive evaluation processes of empathy for pain (Fan and Han, 2008; Sessa and Meconi, 2015; Li et al., 2019), it appears that more mental processing resources to others’ pain were recruited during these time windows for both automatic and controlled processes of empathy for pain. Similarly to previous studies (Sessa et al., 2014; Meng et al., 2020), we also found a main effect of “pain” in LPC amplitude in the pain judgment task, with painful pictures eliciting more positive LPC waves than non-painful pictures. These results may suggest that LPC are sensitive to others’ pain cues, independently of facial skin color and attractiveness. As LPC over the posterior parietal cortical area have been thought to link to a cognitive evaluation component of empathy (Xia et al., 2016; Meng et al., 2019), it appears that processing resources of evaluation of others’ pain were recruited automatically in LPC time windows even though the pain cues in the pain judgment Task were related to attractiveness and skin color. In addition, N170 was not modulated by the blending effects between facial skin color and attractiveness of the faces. About the N170, previous results are regard to the sensitivity of this component for race-specific appearance (Ito and Urland, 2005; Stahl et al., 2008) and the existence of other-race faces elicited the enhanced N170 component. But, the different facial skin colors in this study may not have made participants aware of racial identity differences. This effectively controls for the interference of the irrelevant variable of ethnic identity.

To reduce the influence of the empathy-irrelevant distractor, we calculated differential ERP waveforms between painful and non-painful pictures to reveal the underlying neural processing for empathy for pain, for which the method has been used widely in previous studies (Ibáñez et al., 2011; Meng et al., 2012; Cui et al., 2016). In the present study, we found a significant interaction of “skin color” and “attractiveness” in the differential N2 amplitudes to others’ pain, whereby more attractive dark-skinned faces elicited smaller N2 amplitudes than more attractive fair- and wheatish-skinned faces. However, empathic responses to the less attractive faces were not influenced by skin color. Given that the frontal N2 component is thought to be related to the affective components of empathy for pain (Chen et al., 2012; Luo et al., 2018) and N2 amplitudes have shown to be positively correlated to the degree of empathic responses to others’ pain (Mella et al., 2012; Fabi and Leuthold, 2017), decreased affective empathy is likely to be elicited toward people with more attractive dark-skinned faces. In addition, pain intensity ratings to the more attractive dark-skinned faces were lower than to the more attractive fair- and wheatish-skinned faces. Our results suggest that skin color modulates empathy for pain toward more attractive individuals. Moreover, relative to more attractive people with fair and wheatish skin, empathic responses to pain of more attractive people with dark skin are inhibited.

One possible explanation for the present findings is that empathic responses to others’ pain might be sensitive to their physical condition (Fisher and Ma, 2014). Empathy for pain to healthier (Carrito et al., 2016; Bixley et al., 2018; Dias, 2020) and younger (Tamm et al., 2017) individuals have shown to be reduced. It is possible that people with more attractive dark-skinned faces may be considered healthier and younger than other people, and thus be perceived as having better physical fitness. From an evolutionary perspective, physical fitness has important implications in terms of resisting potential threat and harm (Visconti et al., 2018). Previous work has shown that higher physical fitness is linked to a decreased risk of physical pain (Xiang et al., 2018; Chen et al., 2020), and an antecedent to empathy toward another person is the perception or awareness that the person is in need of help (Bershad et al., 2018). Thus, people with more attractive dark-skinned faces may be perceived as having better physical fitness and being less susceptible to painful feelings; thus, empathic responses to their pain may be inhibited. Interestingly, empathy for less attractive painful faces was not influenced by skin color. Possible explanation for this result would be that the facial attractiveness might influence our process to skin color of faces. Compared more attractive faces, Less attractive faces may not attract too much attention from participants and be processed facial features effectively and fast (Chen et al., 2012). Previous studies on the early perception of attractiveness demonstrated that attractive faces would activate a rapid and automatic perception in a very short time (Sui and Liu, 2009; Rellecke et al., 2011). The facial features of less attractiveness faces are spent more long time for participants to perception (Olson and Marshuetz, 2005). Thus, when participants pay attention to the less attractive faces, empathy for less attractive painful faces was more difficult to be influenced by skin color.

Despite these possible implications, several limitations of the present study should also be addressed. Firstly, both female and male faces were used in the study, and the effects of gender may influence results. Secondly, painful pictures depicted a syringe needle penetrating a model’s cheek. Whether these pictures reflect painful situations in daily life requires further investigation. Finally, the interaction between skin color and attractiveness on empathy for pain was induced experimentally, but the generalizability of the results to real life situations requires further investigation.

## Conclusion

We used pictures of faces within participants’ racial in-group to examine whether empathy for pain is affected by skin color and attractiveness of others’ faces. Results suggested that both behavioral and neural empathic responses to more attractive dark-skinned painful faces are lower relative to those to more attractive fair- or wheat-skinned faces, whereas empathy for less attractive painful faces were not influenced by skin color. Thus, empathy for pain may be influenced by the interplay between others’ skin color and attractiveness.

## Data Availability Statement

The datasets presented in this study can be found in online repositories. The names of the repository/repositories and accession number(s) can be found in the article/Supplementary Material.

## Ethics Statement

The studies involving human participants were reviewed and approved by the Local Research Ethics Committee of Chongqing Normal University. The participants provided their written informed consent to participate in the study.

## Author Contributions

DY: conceptualization, methodology, software, data curation, and writing—original draft preparation. XL: methodology and software. YZ: data curation and writing—original draft preparation. ZL: supervision. JM: conceptualization, methodology, and writing—reviewing and editing. All authors contributed to the article and approved the submitted version.

## Conflict of Interest

The authors declare that the research was conducted in the absence of any commercial or financial relationships that could be construed as a potential conflict of interest.

## Publisher’s Note

All claims expressed in this article are solely those of the authors and do not necessarily represent those of their affiliated organizations, or those of the publisher, the editors and the reviewers. Any product that may be evaluated in this article, or claim that may be made by its manufacturer, is not guaranteed or endorsed by the publisher.

## Footnotes

1
http://stats.idre.ucla.edu/other/gpower/
