# Supplementary material for: Skin Color and Attractiveness Modulate Empathy for Pain: An Event-Related Potential Study
Source: Front Psychol. 2022 Jan 4;12:780633. doi: 10.3389/fpsyg.2021.780633 (PMC8763853; doi:10.3389/fpsyg.2021.780633)
Supplement: Supplementary file 1 [file Data_Sheet_1.doc]

**Supplementary Material**

|  | **Fair faces** | **Wheat faces** | **Dark faces** | ***F*** | ***p*** | **η_p_^2^** |
| --- | --- | --- | --- | --- | --- | --- |
| **Attractiveness** | 4.69±0 .68 | 4.76±0.68 | 4.70±0.69 | 0.233 | 0.792 | 0.45 |
| **Skin color** | 3.50±0.24 | 5.03±0.23 | 6.33±0.28 | **254.134** | **＜0.001** | **0.88** |
| **Emotional valence** | 4.40±0.38 | 4.51±0.39 | 4.43±0.40 | 1.719 | 0.061 | 0.73 |

Supplementary Table 1. Total stimulus material of faces pictures evaluation data (Mean ± SEM). Statistic results were obtained using three-way repeated measures ANOVA of “fair” “wheatish” and “dark”. Significant comparisons (*p* < 0.05) were shown in boldface

|  | **Attractiveness** | **Non-painful** | | | **Painful** | | |
| --- | --- | --- | --- | --- | --- | --- | --- |
|  |  | **Fair skin** | **Wheat skin** | **Dark skin** | **Fair skin** | **Wheat skin** | **Dark skin** |
| **RT (ms)** | **Less** | 670.56 ± 20.83 | 669.97 ± 19.39 | 675.05 ± 20.88 | 685.13 ± 29.46 | 694.76 ± 29.25 | 677.53 ± 23.25 |
|  | **More** | 671.12 ± 22.41 | 658.93 ± 22.99 | 680.54 ± 20.55 | 704.36 ± 27.50 | 689.24 ± 27.96 | 671.96 ± 26.21 |
| **ACC (%)** | **Less** | 98.95 ± 4.16 | 98.25 ± 5.36 | 98.87 ± 4.34 | 98.11 ± 4.12 | 98.01 ± 7.12 | 98.1 ± 5.12 |
|  | **More** | 98.41 ± 5.94 | 98.53 ± 7.21 | 98.43 ± 5.21 | 97.47 ± 7.20 | 97.23 ± 6.78 | 97.61 ± 8.31 |
| **Pain intensity rating** | **Less** | 5.38 ± 0.26 | 5.69 ± 0.27 | 5.13 ± 0.25 | 7.44 ± 0.23 | 6.99 ± 0.28 | 7.39 ± 0.25 |
|  | **More** | 5.72 ± 0.27 | 5.10 ± 0.30 | 5.49 ± 0.28 | 6.92 ± 0.28 | 7.35 ± 0.32 | 6.13 ± 0.22 |
| **Attractive rating** | **Less** | 5.18 ± 0.26 | 5.11 ± 0.23 | 5.25 ± 0.22 | 4.91 ± 0.24 | 5.17 ± 0.25 | 5.18 ± 0.27 |
|  | **More** | 5.62 ± 0.28 | 5.94 ± 0.31 | 5.59 ± 0.28 | 5.90 ± 0.31 | 5.30 ± 0.29 | 5.38 ± 0.28 |
| **Skin color rating** | **Less** | 4.03 ± 0.15 | 4.89 ± 0.25 | 6.58 ± 0.19 | 3.48 ± 0.17 | 4.92 ± 0.20 | 6.70 ± 0.17 |
|  | **More** | 3.31 ± 0.16 | 4.96 ± 0.27 | 6.70 ± 0.14 | 3.22 ± 0.16 | 4.50 ± 0.18 | 6.69 ± 0.18 |
| **Emotional reaction** | **Less** | 4.87 ± 0.20 | 4.50 ± 0.29 | 4.86 ± 0.22 | 4.48 ± 0.20 | 4.32 ± 0.23 | 4.43 ± 0.23 |
|  | **More** | 5.55 ± 0.21 | 5.80 ± 0.18 | 5.39 ± 0.19 | 4.98 ± 0.14 | 4.90 ± 0.13 | 5.13 ± 0.19 |

Supplementary Table 2. Summary of descriptive results of behavioral data (Mean ± SEM)

|  | **Pain** | | | **Attractiveness** | | | **Skin color** | | | **Attractiveness× Pain** | | | **Pain × Skin color** | | | **Attractiveness × Skin color** | | | **Pain ×**  **Attractiveness ×**  **Skin color** | | |
| --- | --- | --- | --- | --- | --- | --- | --- | --- | --- | --- | --- | --- | --- | --- | --- | --- | --- | --- | --- | --- | --- |
|  | ***F*** | ***p*** | **η_p_^2^** | ***F*** | ***p*** | **η_p_^2^** | ***F*** | ***p*** | **η_p_^2^** | ***F*** | ***p*** | **η_p_^2^** | ***F*** | ***p*** | **η_p_^2^** | ***F*** | ***p*** | **η_p_^2^** | ***F*** | ***p*** | **η_p_^2^** |
| **N1** | **5.23** | **0.032** | **0.19** | 1.35 | 0.258 | 0.55 | **27.27** | **<0.001** | **0.71** | 0.55 | 0.465 | 0.02 | 1.95 | 0.156 | 0.78 | 2.95 | 0.700 | 0.11 | 0.18 | 0.819 | 0.01 |
| **N170** | 0.23 | 0.638 | 0.01 | 0.02 | 0.902 | <0.01 | **6.77** | **0.005** | **0.38** | 0.36 | 0.554 | 0.02 | 0.98 | 0.382 | 0.04 | **8.58** | **0.001** | **0.27** | 3.31 | 0.550 | 0.23 |
| **N2** | 3.06 | 0.094 | 0.12 | 0.29 | 0.598 | 0.01 | **5.86** | **0.008** | **0.20** | 2.62 | 0.119 | 0.10 | 2.17 | 1.310 | 0.09 | 1.42 | 0.254 | 0.06 | **4.16** | **0.023** | **0.15** |
| **P2** | 0.42 | 0.638 | 0.01 | 0.71 | 0.409 | 0.03 | 0.49 | 0.615 | 0.02 | 1.40 | 0.249 | 0.06 | 0.34 | 0.687 | 0.01 | 0.05 | 0.937 | <0.01 | 3.21 | 0.051 | 0.11 |
| **P3** | **10.33** | **0.008** | **0.27** | 0.01 | 0.913 | <0.01 | 0.24 | 0.789 | 0.01 | 3.09 | 0.092 | 0.12 | 0.57 | 0.566 | 0.02 | 0.04 | 0.950 | <0.01 | 0.49 | 0.591 | 0.02 |
| **LPC** | **18.47** | **<0.001** | **0.45** | 0.72 | 0.404 | 0.03 | 1.13 | 0.325 | 0.05 | 2.27 | 0.145 | 0.09 | 1.48 | 0.239 | 0.06 | 0.50 | 0.605 | 0.02 | 1.05 | 0.359 | 0.04 |

Supplementary Table 3. Statistic results of amplitudes of the dominant ERP components were obtained using three-way repeated measures ANOVA of “skin color” (fair, wheatish, and dark), “attractiveness” (more attractive, less attractive), and “pain” (painful, non-painful). Significant comparisons (*p* < 0.05) were shown in boldface
